# Pharmacologic management of metabolic and alcohol-associated liver disease

**DOI:** 10.20517/mtod.2026.29

**Published:** 2026-06-16

**Authors:** Mangesh Pagadala, Sajid Jalil, Nicholas Dunn, Ashwani K. Singal

**Affiliations:** 1 Liver Institute, Methodist Dallas Medical Center, Dallas, TX 75203, USA; 2 Division of Gastroenterology and Hepatology, Stanford University School of Medicine, Palo Alto, CA 94305, USA; 3 Pembroke Hill School, Kansas City, MO 64112, USA; 4 Division of Gastroenterology Hepatology and Nutrition, University of Louisville School of Medicine, Louisville, KY 40202, USA

**Keywords:** MetALD, steatotic liver disease, MASLD, alcohol-associated liver disease, GLP-1 receptor agonists, FGF21 analogs, thyroid hormone receptor-βagonists, alcohol use disorder

## Abstract

Metabolic and alcohol-associated liver disease (MetALD) is an emerging phenotype within the steatotic liver disease spectrum, characterized by cardiometabolic risk factors coexisting with alcohol exposure, resulting in synergistic liver injury and fibrosis progression. Therapeutic development remains limited because most steatotic liver disease trials exclude patients with ongoing alcohol use. In contrast, alcohol-associated liver disease (ALD) trials have focused primarily on severe alcohol-associated hepatitis. Current management of patients with MetALD relies on an integrated approach that simultaneously controls alcohol use and cardiometabolic risk. In clinical practice, for patients with MetALD and ongoing alcohol use, therapies aimed at controlling alcohol use remain most critical, given the faster, more progressive disease course related to alcohol as compared to metabolic liver injury. Given the dynamic nature of alcohol intake and metabolic risk factors, longitudinal monitoring of disease stage with noninvasive fibrosis tests is essential. Liver-directed therapies with efficacy in metabolic dysfunction-associated steatotic liver disease (MASLD), including incretin-based agents, fibroblast growth factor 21 analogs, peroxisome proliferator-activated receptor agonists, and thyroid hormone receptor beta agonists, may benefit selected MetALD patients. Several agents may modulate both metabolic pathways and alcohol consumption through central reward mechanisms. Specific pharmacotherapies targeting alcohol use (acamprosate, naltrexone) combined with structured psychosocial interventions are effective in controlling alcohol use. Given the lack of dedicated clinical trials in MetALD patients, we synthesized data from clinical trials in MASLD and ALD. We propose adapting these data to inform the design of future clinical trials in patients with MetALD.

## Graphical Abstract

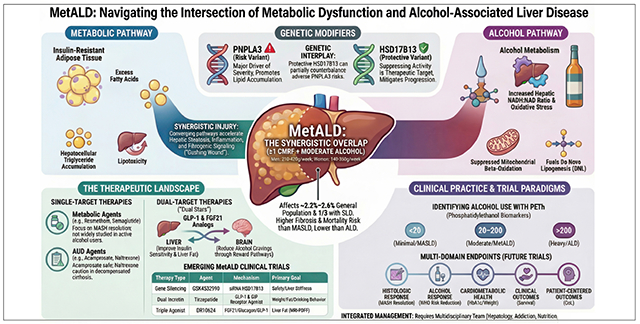

## INTRODUCTION

Metabolic dysfunction and alcohol-associated liver disease (MetALD) is a distinct subset of steatotic liver disease (SLD)^[1]^. MetALD is diagnosed in the presence of ≥ 1 cardiometabolic risk factor (CMRF) and 210-420 g/week of pure ethanol for men and 140-350 g/week for women^[1,2]^. CMRF in the context of SLD includes obesity or increased waist circumference, hypertension, type 2 diabetes mellitus, low high-density lipoprotein, and elevated triglycerides. The prevalence of MetALD from cohort studies is estimated at 2.2%-2.6% in the general population^[3,4]^ and 31.8%-33.2% of individuals with SLD^[5,6]^.

MetALD results from the synergistic interaction between metabolic dysfunction and alcohol. The two etiologies share underlying mechanisms, including dysregulated lipid and bile acid metabolism. As this is a new entity introduced under the SLD nomenclature, emerging data on the natural history indicate that individuals with MetALD have an intermediate risk of liver fibrosis, decompensation, and mortality, higher than metabolic dysfunction-associated steatotic liver disease (MASLD) but lower than alcohol-associated liver disease (ALD)^[7]^. Data are also emerging on the accuracy and cut-off points of serum and imaging-based non-invasive tests for assessing fibrosis in patients with MetALD.

In this narrative review, we will focus on the management of patients with MetALD. Simply utilizing treatments for MASLD and ALD independently, rather than within an integrated framework, would be a band-aid solution for a gushing wound. Hence, both risk factors need to be managed simultaneously with lifestyle modification and pharmacological therapies. Given the accelerated risk of progression with alcohol use compared to metabolic risk factors, the priority should be controlling alcohol use. Potential inaccuracy in self-reported alcohol use should be recognized, which underscores the role of biomarkers of alcohol intake to substantiate the information. Further, given the dynamic nature of the risk factors, especially alcohol use, periodic assessment of risk factors and the stage of liver disease should be performed^[8]^.

A literature search was conducted in PubMed and ClinicalTrials.gov databases to identify studies related to MetALD, SLD, MASLD, MASH, ALD, alcohol use disorder, glucagon-like peptide 1 (GLP-1) receptor agonists, fibroblast growth factor 21 (FGF21) analogs, and thyroid hormone receptor-beta (THR-β) agonists. The search and manuscript preparation were completed, while the literature review included relevant preclinical studies, clinical trials, observational studies, practice guidelines, and review articles published between January 2010 and March 2026. Reference lists of selected articles were also manually screened to identify additional relevant studies. Articles were selected based on relevance to the pathophysiology, diagnosis, pharmacologic management, and clinical outcomes of MetALD and related SLD. Non-English articles, duplicate publications, and studies not directly relevant to MetALD, MASLD, ALD, or alcohol use disorder therapeutics were excluded.

## THERAPEUTIC LANDSCAPE AND STRATEGIES TO MANAGE MetALD

### Pathophysiologic rationale

Alcohol metabolism increases the hepatic NADH/NAD ratio, promotes oxidative stress, which in turn suppresses mitochondrial beta-oxidation, and enhances *de novo* lipogenesis (DNL)^[2,9]^. In parallel, insulin-resistant adipose tissue increases lipolysis and releases excess non-esterified fatty acids into the portal circulation, contributing to hepatocellular triglyceride accumulation, lipotoxicity, and downstream endoplasmic reticulum and mitochondrial stress^[10]^. The synergistic effects of the two risk factors augment hepatic steatosis, inflammation, and fibrogenic signaling in MetALD. Several observational studies have shown that individuals with MetALD have a higher prevalence of steatohepatitis, more advanced fibrosis, and an increased risk of hepatic decompensation compared with those exposed to either metabolic risk factors or alcohol alone^[2,11]^. Together, these data support a therapeutic framework for MetALD that targets the metabolic and inflammatory mechanisms driving liver injury, while alcohol use disorder is addressed in parallel through addiction-focused interventions.

Mechanistic plausibility, while necessary, is not sufficient to establish clinical efficacy. As in other areas of medicine, translation from bench to bedside in hepatology is associated with a high failure rate, as most phase 2 and 3 trials prioritize clinical outcomes over direct interrogation of underlying mechanisms. This limitation applies to all therapeutic targets discussed below.

## MANAGEMENT OF CARDIO-METABOLIC RISK FACTORS

### Non-pharmacologic management

Lifestyle modifications are a major part of treating MetALD. A weight-loss goal of ≥ 7%-10% is recommended to achieve beneficial effects on steatosis and fibrosis^[12]^. In the absence of weight loss, hepatic and cardiometabolic benefits can also be achieved by improving diet quality and physical activity^[13]^. Lifestyle interventions should adopt an approach that addresses diet, exercise, smoking, alcohol intake, and sleep. Mediterranean-style diets rich in unsaturated fats and fiber are recommended^[14,15]^. Structured moderate-to-vigorous aerobic exercise for 150-240 min per week combined with resistance training reduces steatosis and preserves lean body mass^[5]^. For individuals who do not achieve meaningful weight loss with lifestyle and pharmacologic therapy, bariatric surgery may be considered^[16]^. In a meta-analysis, bariatric therapies in MASH showed a 72% reduction in intrahepatic fat at 6 months and a 50% reduction in NAFLD activity score (NAS) at 36-60 months^[17]^. CMRF should be controlled by optimizing blood pressure, blood sugar, lipids, and smoking cessation^[15]^. High-intensity statins are the primary therapy for dyslipidemia, are safe, and should be used whenever indicated^[18]^.

### Pharmacologic management

The drug development landscape for SLD in MASLD patients is extensive [Table 1]. This has led to FDA approval of resmetirom, a THR-β agonist, in March 2024, and semaglutide, a GLP-1 receptor agonist, in August 2025, for the treatment of MASH patients with fibrosis stages 2-3^[19,20]^. However, these drugs were not studied in patients with MetALD. On the other hand, the drug development landscape in ALD has been relatively less advanced and has focused mainly on the severe form of alcohol-associated hepatitis (AH). Clearly, there is an unmet clinical need to assess the safety and efficacy of available drugs and to develop new targets for the population with MetALD. Further, divergent nutritional phenotypes in MetALD patients with obesity and CMRF on one side and malnutrition with nutrient deficiencies from alcohol use on the other (obese sarcopenia) are a challenge^[21]^. Drawing on the drug development landscape, we review therapeutic targets for patients with MetALD.

### Drugs modulating insulin resistance

#### Incretin based therapies (GLP-1 receptor agonists and dual GLP1/GIP agonists)

The clinical trials on GLP-1 receptor agonists have been mainly performed in patients with MASLD and MASH, and excluded patients with excess alcohol use [Table 1]. Incretin-based agonists to receptors, including GLP-1 (exenatide, liraglutide, and semaglutide), dual GLP-1/gastric inhibitory polypeptide or GIP (tirzepatide), or GLP-1 and glucagon receptor (survodutide), and triple agonists (retatrutide), have gained increasing attention^[22]^. These agents promote weight loss, improve insulin sensitivity, and reduce systemic and hepatic inflammation. In addition, emerging data suggest a role in modulating alcohol intake through central appetite and reward pathways^[23,24]^. GLP-1 receptor agonists exert central and peripheral effects through the gut-brain axis, promoting satiety, delaying gastric emptying, and reducing caloric intake, and improve mitochondrial function by suppressing DNL^[25]^.

In a phase 2 randomized trial of 190 patients with biopsy-proven MASH, once-daily semaglutide administered for 52 weeks resulted in significantly higher rates of MASH resolution without worsening fibrosis compared with placebo^[26]^. More recently, results from the phase 3 ESSENCE trial demonstrated that 72 weeks of semaglutide therapy led to MASH improvement in 62.9% of treated patients, compared with 34.1% with placebo, and fibrosis improvement in 37%, compared with 22.5% with placebo (*P* < 0.0001 for both endpoints)^[27]^. Dual incretin therapy with tirzepatide has also demonstrated a reduction in hepatic steatosis, as measured by imaging-based liver fat quantification at one year, though histologic outcomes remain under investigation^[28]^.

#### FGF21 analogs

FGF21 is a hormone with endocrine, autocrine, and paracrine activity. It is produced predominantly by the liver, adipose tissue, and other organs^[29,30]^. FGF21 exerts its metabolic effects primarily by promoting lipid catabolism and increasing insulin sensitivity, thereby enhancing lipolysis, fatty acid mitochondrial beta-oxidation, and energy dissipation. It also modulates appetite via a central mechanism and decreases preference for sugar and alcohol. Collectively, these effects reduce hepatic lipotoxicity, inflammation, and fibrogenesis, supporting its role in SLD and steatohepatitis^[31,32]^.

In phase 2 studies, the FGF21 analog efruxifermin reduced the hepatic fat fraction. Among patients with MASH cirrhosis, 25% achieved MASH resolution, including at least one stage of fibrosis improvement^[33]^. In a recent meta-analysis of eight randomized trials involving 963 MASH patients, FGF21 analogs (efruxifermin, pegbelfermin, and pegozafermin) were superior to placebo in improving liver fibrosis by ≥ 1 stage (27% *vs*. 14%), with a pooled risk ratio of 1.83 (1.27-2.62). FGF21 showed class heterogeneity. For example, a subgroup analysis on at least two-point reduction in NAS without worsening of fibrosis showed the lowest efficacy with pegozafermin (RR = 2.48; 95%CI: 1.57-3.93; *P* = 0.0001) and the highest with efruxifermin (RR = 3.50; 95%CI: 2.01-6.08; *P* < 0.00001)^[34]^.

#### Peroxisome proliferator-activated receptor agonists

Peroxisome proliferator-activated receptor (PPAR) agonists target lipid metabolism, insulin sensitivity, inflammation, and fibrogenesis^[35]^, to enhance hepatic fatty acid oxidation. Specifically, PPAR β and δ receptors promote thermogenesis and reduce lipogenesis, while PPAR γ improves insulin sensitivity. These beneficial effects modulate inflammatory and fibrotic signaling^[36,37]^.

Lanifibranor, a pan-PPAR agonist, in a phase 2b trial (NATIVE trial) among MASH patients, showed a ≥ 2-point reduction in the NAS in 55% of patients receiving lanifibranor *vs*. 33% of patients receiving placebo. In treated patients, NASH resolved in 49% and fibrosis improved by at least 1 stage in 48%.

Saroglitazar, a dual PPAR α γ agonist, improved steatosis and fibrosis and additionally led to decreased cardiovascular risk in MASH^[38,39]^. Pioglitazone, a selective PPARγ agonist, has also demonstrated improvements in steatosis and inflammation, with benefits for fibrosis and MASH resolution.

#### Sodium glucose co-transporter −2 inhibitors

Sodium glucose co-transporter −2 (SGLT2) inhibitors improve glucose control and may promote weight loss. In clinical trials, Ipragliflozin, compared to placebo, improved liver outcomes in diabetic patients with MASH, including fibrosis^[40]^. In a meta-analysis of randomized controlled trials in MASH patients, SGLT2 inhibitors were associated with improvements in steatosis and liver fibrosis^[41]^.

### Drugs targeting de-novo-lipogenesis

#### THR-β agonists

THR-β is a nuclear receptor involved in hepatic fatty acid β-oxidation, mitochondrial biogenesis, and cholesterol clearance. Because THR-β is predominantly expressed in the liver, agonism of THR-β improves hepatic metabolism without the extrahepatic effects of THR-α^[42]^.

In the MAESTRO NASH trial in patients with biopsy-proven NASH with F1b to F3 fibrosis, resmetirom 80 and 100 mg at 52 weeks achieved resolution of NASH without worsening of fibrosis in 25.9% and 29.9%, respectively, compared with 9.7% in the placebo group. Improvement in fibrosis by at least one stage was observed in 24.2% and 25.9% of patients receiving 80 and 100 mg, respectively, compared with 14.2% of patients receiving placebo. Furthermore, there was a significant reduction in low-density lipoprotein cholesterol in the resmetirom arms but not in the placebo arm.

#### FXR agonists

These agonists target bile acid-mediated signaling pathways that regulate lipid and glucose metabolism, insulin sensitivity, and downstream metabolic and inflammatory pathways^[43]^.

Several non-bile acid Farnesoid X receptor (FXR) agonists have shown encouraging results in early-phase studies. For example, cilofexor improved hepatic fibrosis without worsening steatohepatitis when combined with the acetyl-CoA carboxylase (ACC) inhibitor firsocostat in a phase 2 study^[44]^. Tropifexor demonstrated dose-dependent reductions in liver enzymes and hepatic fat fraction in a randomized phase 2 trial in patients with MASH^[45]^. Vonafexor, evaluated in the LIVIFY phase 2a trial, was generally well tolerated and associated with significant reductions in liver fat, liver enzymes, and body weight as assessed by magnetic resonance imaging and biochemical markers^[46]^.

#### Inhibitors of DNL and triglyceride synthesis

Key enzymatic targets of DNL include fatty acid synthase (FAS), ACC, ATP citrate lyase, and diacylglycerol acyltransferase (DGAT), which together regulate fatty acid synthesis and triglyceride formation in hepatocytes^[47]^.

Denifanstat, an oral FAS inhibitor, demonstrated histologic efficacy in the phase 2b FASCINATE 2 trials in patients with F2-3 MASH^[48]^. At 52 weeks, patients treated with denifanstat, compared with placebo, achieved a two-point or greater reduction in NAS without worsening of fibrosis in 38 percent *vs*. 16 percent; resolution of MASH in 26 percent *vs*. 11 percent; fibrosis regression of at least one stage in 49 percent *vs*. 13 percent; and reduction in liver fat on magnetic resonance imaging proton density fat fraction (MRI-PDFF) in 31 percent *vs*. 10 percent. In addition, denifanstat rapidly lowered tripalmitin, a biomarker of DNL. DGAT2 inhibitors, such as ervogastat, have demonstrated reductions in liver fat in short phase 2 studies, either as monotherapy or in combination regimens^[49]^.

### Genetic modifiers of hepatic lipid accumulation and treatment response

The Patatin-Like Phospholipase Domain Containing 3 (*PNPLA3*) and *HSD17B13* genetic polymorphisms are most commonly established in the development and severity of SLD, including MetALD^[50]^. The PNPLA3 I148M variant is associated with increased steatosis, progression of fibrosis, and greater severity of SLD, including MetALD. In contrast, HSD17B13 loss- of-function variants appear protective against progressive liver injury. When both variants are present, the protective effect of HSD17B13 may partially attenuate the adverse risk associated with PNPLA3^[51,52]^. The therapeutic targeting of *HSD17B13* is currently being evaluated in a Phase 2 clinical trial (NCT06613698) in patients with MetALD.

Currently, several promising agents are advancing through clinical development and have the potential to be approved treatment options for MASH. These include FGF21 analogs such as efruxifermin and pegozafermin^[33]^, PPAR agonist lanifibranor^[53]^, and Survodutide^[54]^. Of these, resmetirom and efruxifermin are also being evaluated in patients with MASH cirrhosis (F4). These trials are designed to assess the safety and efficacy of the intervention as a pathway to regulatory drug approval, and they vary across countries depending on the respective regulatory frameworks, alcohol use patterns, disease prevalence, and healthcare access.

### Combination therapeutic approaches

Given multiple therapeutic targets with distinct mechanisms, combination therapy represents a promising strategy to enhance therapeutic efficacy. Several clinical trials are currently underway that use combinations of different agents [Table 2]. Of particular interest are combinations of GLP-1 receptor agonists with FGF21 analogs (NCT05016882), the SGLT2 inhibitor empagliflozin with PPAR agonists (NCT04976283), and with GLP-1 receptor agonists (NCT04639414).

### Gut-liver axis targeted therapies

Despite recognition of the role of gut dysbiosis in MASLD, clinical translation remains limited. In a randomized controlled trial, fecal microbiota transplantation (FMT) in MASLD patients showed a trend toward lower serum triglyceride levels but no change in hepatic fat content or metabolic parameters at 12 weeks^[55]^.

Overall, the emerging therapeutic landscape for MetALD suggests that no single agent is likely to address the full spectrum of underlying processes in this population. Incretin-based therapies currently have the most established clinical evidence and may offer benefits for both metabolic dysfunction and alcohol-related behaviors. FGF21 analogs are also of particular interest for their potential dual metabolic and alcohol-modulating effects. However, dedicated studies in MetALD are lacking for both GLP-1 and FGF21 agonists. THR-β agonists such as resmetirom appear promising for fibrosis-directed therapy, while PPAR agonists may have a role in carefully selected patients. Given the heterogeneity and dynamic nature of MetALD, future management will likely require individualized, potentially combination-based approaches targeting both metabolic dysfunction and alcohol use.

## THERAPIES FOR ALCOHOL USE DISORDER

### Non-pharmacologic management

Abstinence improves long-term outcomes in patients with liver disease and comorbid alcohol use disorder (AUD)^[56]^. Evidence-based nonpharmacological treatments for AUD include motivational interviewing with brief intervention to counsel patients on alcohol-related harms, cognitive-behavioral therapy, motivational enhancement therapy, contingency management approaches, couples and family counseling, and 12-step facilitation therapy^[57]^. Psychosocial interventions are more effective when combined with pharmacological therapies^[58]^.

### Pharmacological therapies

FDA-approved medications for alcohol use disorder (MAUD) include disulfiram, naltrexone, and acamprosate^[59-61]^. Nalmefene is approved by the European Medicines Agency but is not available in the US for the treatment of AUD^[59,62]^. [Table 3].

Acamprosate is not metabolized in the liver and is safe in patients across the spectrum of ALD. However, randomized trials in patients with ALD are lacking. In a small, open-label phase II trial of 12 patients with ALD, acamprosate reduced craving scores in all participants with no adverse events^[63]^. A dose reduction is necessary in patients with renal impairment. Baclofen, gabapentin, and topiramate are additional options for patients unable to take acamprosate or naltrexone, as they are not significantly metabolized in the liver, though they require renal dose adjustment.

### Dual targets

#### Incretin-based therapies

Observational data suggest that incretin-based therapies may reduce alcohol intake in some individuals with obesity and AUD^[64,65]^. In a retrospective analysis of 153 obese individuals, 106 patients treated with GLP-1-based therapies (semaglutide and tirzepatide) reported a reduction in alcohol intake after treatment initiation, compared with 47 control patients not receiving incretin therapy^[66]^. Similar observational findings have been reported in other retrospective cohorts, suggesting a potential class effect^[64,67]^. These observational studies relied on propensity score matching to account for differences between treatment groups and did not account for unmeasured confounding, reverse causation, healthy-user bias, or publication bias. These limitations constrain causal interpretation of these observations.

Clinical trials that achieve comparability by allocating treatment groups randomly across participants are needed to corroborate these encouraging observational findings.

Randomized controlled trials are limited by sample size, varying interventions and durations, and inconsistent results: two studies showed benefit, while one showed no benefit^[23]^. However, none of these three trials recruited patients with liver disease.

#### FGF21 analogs

FGF21 is uniquely positioned as alcohol increases endogenous circulating FGF21 levels. FGF21 signaling may additionally reduce alcohol consumption through central reward pathways^[68-70]^. Furthermore, human genetic analyses using Mendelian randomization provide strong causal evidence that enhanced FGF21 signaling reduces alcohol consumption and the risk of alcohol use disorder^[71]^. Although there is strong experimental data, clinical trials are needed to examine the benefit of FGF21 agonists in reducing alcohol intake in patients with ongoing alcohol use.

#### THR-β agonists

THR-β agonists reduced steatosis and liver injury in preclinical ALD models, but clinical data in patients with alcohol use remains lacking^[72]^.

#### PPAR agonists

Preclinical studies suggest PPAR agonists may influence alcohol intake^[73,74]^. The only available human study using pioglitazone in 16 subjects reported increased alcohol craving and risk of myopathy, leading to early termination of the study^[75]^. In contrast, the PPARα agonist fenofibrate has shown reduced voluntary alcohol consumption in rodent models^[76-78]^. Mechanistic studies suggest modulation of GABAergic interneurons within the amygdala^[74]^. Taken together, signals of increased alcohol craving with pioglitazone and the limited human data on dual agonists in active alcohol use indicate that well-designed studies in carefully selected patients are needed to substantiate the role of these drugs for alcohol use.

#### Inhibitors of DNL and triglyceride synthesis

In the absence of clear effects on alcohol related metabolic pathways, these agents may have a limited role in patients with active alcohol use and may be better suited for individuals with MetALD who have achieved sustained reduction or remission of alcohol intake, pending dedicated clinical studies.

### Drugs targeting gut-liver-brain axis

Chronic alcohol use impairs the intestinal barrier and alters gut microbiota composition^[79]^. Bacterial products penetrate the impaired intestinal barrier, triggering hepatic and systemic inflammation and microbiome changes that disrupt the enterohepatic circulation of bile acids^[80]^. In a randomized placebo-controlled trial involving 46 patients with moderate AH, *Lactobacillus rhamnosus* GG supplementation (*n* = 22) compared to placebo resulted in significant improvements in model for end-stage liver disease (MELD) score at 1 month and reduced alcohol consumption at 6 months^[81]^. In a randomized phase 1 trial on 20 patients with ALD cirrhosis and AUD, FMT enema (*n* = 10) compared to placebo reduced alcohol craving and consumption, with favorable microbial changes^[82]^. Although promising, these two high-quality trials of interventions to restore gut dysbiosis remain proof-of-concept. Further, none of these trials included patients with MetALD. Clearly, future trials with larger sample sizes in the MetALD population are needed before these results can inform clinical practice.

### Gene editing therapy for AUD

Gene-editing therapies, such as clustered regularly interspaced short palindromic repeats (CRISPRs), may be used to modify genes encoding alcohol dehydrogenase (ADH) and aldehyde dehydrogenase (ALDH), enzymes involved in alcohol metabolism^[80,83]^.

### Current landscape of clinical trials in MetALD

**Figure 1.**
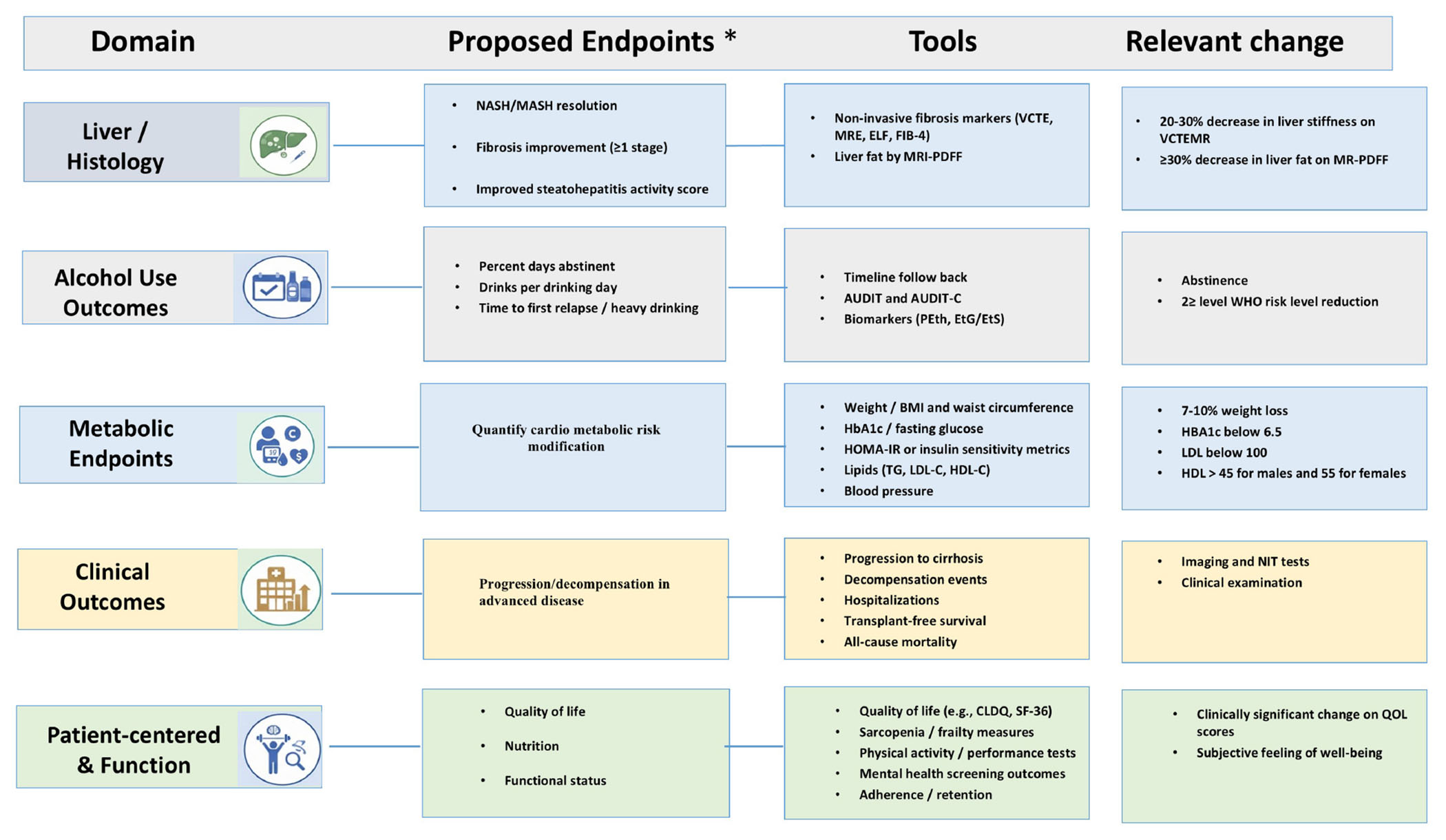
Proposed Endpoint Domains and Measures for MetALD Clinical Trials. *Integrated efficacy monitoring algorithm (month 12 and annually). Histologic response: MASH resolution without worsening of fibrosis, and/or fibrosis improvement ≥ 1 stage without worsening of steatohepatitis. NIT: ≥ 30% reduction in MRI-PDFF; LSM ↓ ≈ 20% from baseline and/or improvement in biomarker NIT from baseline (ELF, FIB-4) from baseline. Alcohol-use response: Abstinence and ≥ 2 WHO risk level reduction on alcohol use (abstinence is no alcohol use; low level is 1-20 g/d for women and 1-30 g/d for men; moderate level is 21-40 g/d for women and 31-60 g/d for men; high level is 41-60 g/d for women and 61-100 for men; and very high level is > 60 g/d for women and >100 g/d for men). Cardio metabolic response: weight ↓ ≥ 5%-10% or waist ↓; HbA1c ↓ ≥ 0.5% or improved fasting glucose; HOMA-IR ↓; TG/LDL-C ↓, HDL-C ↑; BP ↓ or controlled. Clinical outcomes: no progression to cirrhosis; absence of decompensation (ascites, variceal bleeding, encephalopathy); reduced hospitalizations; improved transplant-free survival; reduced mortality. Patient-centered outcomes: QoL ↑ (CLDQ/SF-36); sarcopenia/frailty ↓; physical performance ↑; mental health scores ↑; adherence/retention ↑. BMI: Body mass index; CLDQ: Chronic Liver Disease Questionnaire; ELF: Enhanced Liver Fibrosis test; EtG: ethyl glucuronide; EtS: ethyl sulfate; FIB-4: Fibrosis-4 index; HbA1c: hemoglobin A1c; HDL-C: high-density lipoprotein cholesterol; HOMA-IR: Homeostasis Model Assessment of Insulin Resistance; LDL-C: low-density lipoprotein cholesterol; MASH: metabolic dysfunction-associated steatohepatitis; MRE: magnetic resonance elastography; MRI-PDFF: magnetic resonance imaging-proton density fat fraction; NASH: nonalcoholic steatohepatitis; PACS: Penn Alcohol Craving Scale; PEth: phosphatidylethanol; SF-36: 36-Item Short Form Health Survey; TG: triglycerides; VCTE: vibration-controlled transient elastography.

The therapeutic landscape for MetALD is evolving from treating MASH and ALD separately to integrated management targeting both risk factors [Figure 1]^[84,85]^. Management of MetALD focuses on alcohol cessation and optimization of CMRF. However, several features warrant consideration when designing clinical trials in MetALD. Given the unique nature of this condition, accurate information on alcohol use, complemented by phosphotidylethanol (PEth) testing, will be required to define the population. Further, we need to recognize that alcohol use limits to identify MetALD are based on consensus and have not been validated across populations with divergent patterns of alcohol use. Understanding how variations in drinking patterns across cultures and countries may produce different pathophysiologic consequences and require different therapeutic strategies. Beyond overall safety assessment, these trials would need to examine the interaction of the drug with other medications used for managing metabolic comorbidities and with ongoing alcohol use. For example, resmetirom interacts with statins and clopidogrel when prescribed simultaneously to patients with MASLD and MetALD. Hence, it is recommended to limit the statin dose to a maximum of 20 mg (simvastatin and rosuvastatin) and 40 mg (pravastatin and atorvastatin), and to reduce the resmetirom dose by 20 mg in patients taking clopidogrel. As gemfibrozil is a strong inhibitor of the CYP pathway involved in resmetirom metabolism, it is contraindicated for the treatment of dyslipidemia in patients taking resmetirom. Similar interactions are not known for semaglutide or acamprosate. Studies would also need to assess sarcopenia and its interaction with drug efficacy and pharmacokinetics. The optimal cut-off for non-invasive tests for fibrosis assessment to select the patient population and identify clinically meaningful changes at follow-up would need to be determined to inform pathways for drug approval in patients with MetALD.

An ideal pharmacological approach to treat MetALD patients would be ‘dual targets’, that are effective against SLD and reducing alcohol use. Of the potential targets discussed earlier, GLP-1 and FGF21 agonists are the best candidates for clinical trials in MetALD, as these agents have the strongest evidence of metabolic benefits and the potential to reduce alcohol use [Table 4]. Given the challenges of defining this population and its underrepresentation in clinical drug development, we suggest that ongoing trials in the MASLD and ALD space should measure ongoing alcohol use to perform subgroup analyses in MetALD patients, such as the MAESTRO NASH trial. In a subgroup of 75 patients meeting alcohol use and PEth criteria for MetALD, improvements in fibrosis and MASH were observed, similar to MASH patients^[19,42]^. These data, based on post-hoc analysis of the trial in a small sample of MetALD patients, do suggest a potential role for THR-β agonists, although dedicated trials to examine this hypothesis are needed before their use in practice for patients with MetALD. Taken together, resmetirom, incretin-based therapies, and FGF21 analogs represent promising therapeutic agents that have yet to be fully evaluated in clinical trials.

## CURRENT THERAPEUTIC APPROACH TO MANAGE METALD

MetALD liver injury creates a heterogeneous population in whom sustained abstinence is necessary but rarely sufficient. Therefore, to target both alcohol use and metabolic dysfunction, current management focuses on a combination of lifestyle modifications, management of CMRF, and pharmacotherapy [Table 5]^[86]^. The interaction between two risk factors is sometimes counterintuitive in the management of patients with MetALD. For example, individuals undergoing bariatric surgery, especially Roux-en-Y gastric bypass surgery for weight loss, can trigger new-onset or worsen ongoing alcohol use^[87]^.

**Figure 2.**
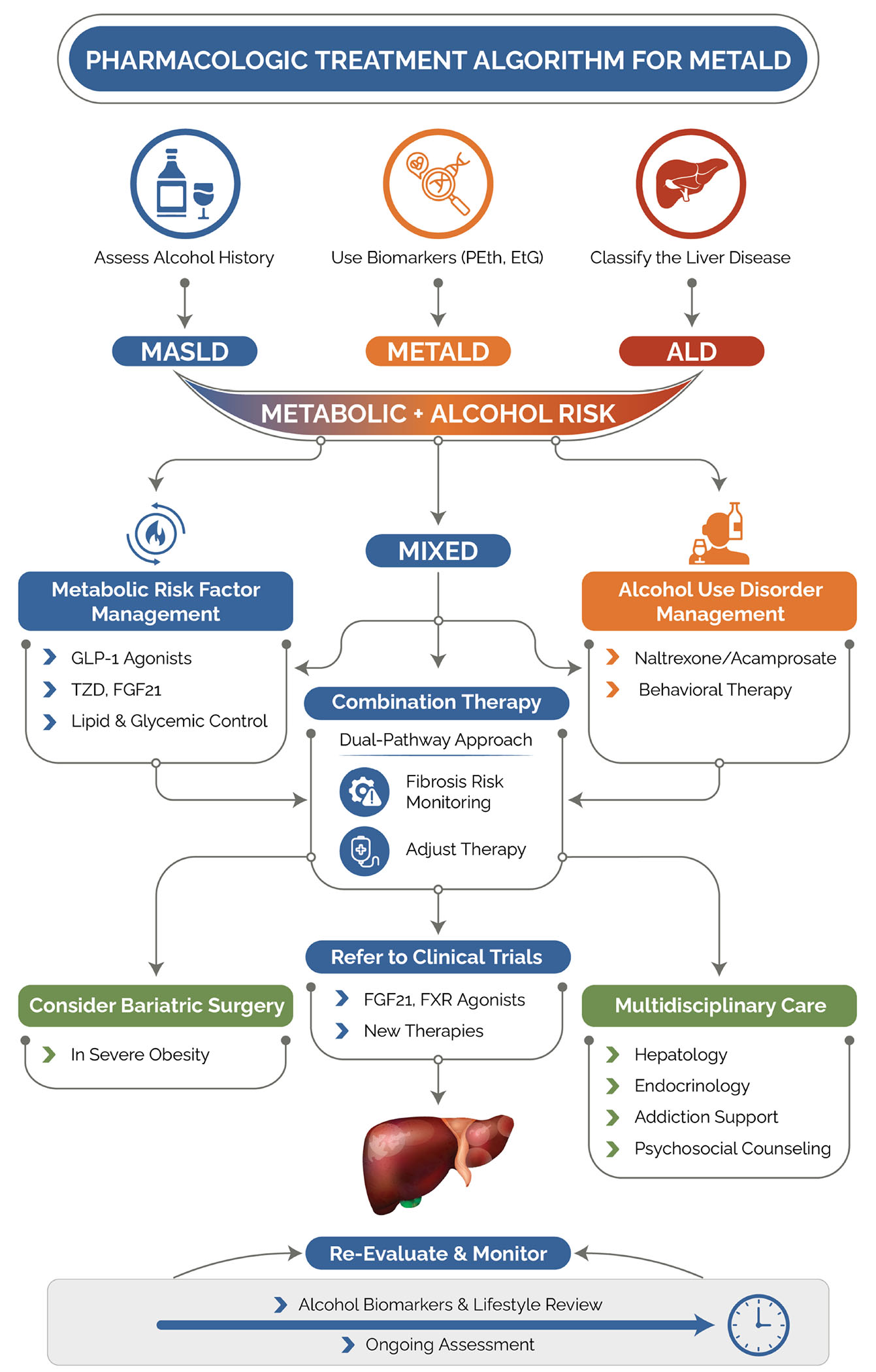
Proposed algorithm for management of patients with MetALD in clinical practice. ALD: Alcohol associated liver disease; EtG: ethyl glucuronide; FGF: fibroblast growth factor; GLP: glucagon-like peptide; MASLD: metabolic dysfunction-associated steatotic liver disease; MetALD: metabolic dysfunction and alcohol associated liver disease; PEth: phosphatidylethanol; T2DM: type 2 diabetes mellitus.

An algorithm for MetALD management involves targeting metabolic risk factors and alcohol [Figure 2]. The first step is to identify the current level of alcohol use using self-reported information (AUDIT-C or timeline follow-back tools), complemented with whole blood PEth level (ng/mL), a biomarker of alcohol metabolism with a window of detection of 3-4 weeks from last alcohol use^[88]^. A PEth level < 20 (minimal or no alcohol use) is consistent with MASLD, 20-200 (moderate alcohol use) is consistent with MetALD, and > 200 (heavy alcohol use) is consistent with ALD^[89]^.

Controlling alcohol use is the most critical factor in patients with active ongoing use, as alcohol-induced injury is more progressive than metabolic liver injury. As the data on safe alcohol use in SLD patients remain unknown, the current recommendation is to abstain completely. For patients abstaining for 3-12 months (early recovery) or more than 12 months (stable recovery) who still have significant fibrosis (F2 or higher), the focus should be on liver-targeted treatments and control of metabolic comorbidities (https://www.niaaa.nih.gov/research/niaaa-recovery-from-alcohol-use) While awaiting safety data for drug therapy in MetALD cirrhosis patients, those with advanced fibrosis (F2-F3) should be considered for treatment with resmetirom or a GLP-1RA. Shared decision-making between the patient and the provider is critical, considering the route of administration and CMRF. For example, a patient with metabolic-predominant disease (diabetes mellitus, BMI > 35, and atherosclerotic disease) is better treated with a GLP-1 receptor agonist. Patients with MetALD who have F0-1 fibrosis stage or with cirrhosis do not currently have specific FDA-approved pharmacotherapies. A multidisciplinary, stepwise approach integrating hepatology, endocrinology, addiction medicine, nutrition, and mental health services is essential^[16]^. On follow-up, monitoring should include non-invasive tests for liver disease; alcohol use using the timeline follow-back tool and PEth test; metabolic comorbidities; nutritional assessment; and quality of life [Figure 2]. Continuing to re-evaluate patients for these outcomes can maximize the effectiveness of treatment for a changing phenotype with changes in alcohol use in MetALD.

## SAFETY CONSIDERATIONS

A structured approach to safety reporting in MetALD should address patient selection, contraindications, monitoring, and risk stratification, given the overlap among liver injury, ongoing alcohol exposure, and metabolic comorbidity in this population. Risk stratification should identify patients who need closer surveillance during therapy, particularly those with ongoing alcohol use, advanced fibrosis, cardiometabolic disease, fluid retention, or baseline muscle loss, rather than relying on a single disease label.

PPAR agonists such as lanifibranor and saroglitazar may be less suitable for patients at risk of fluid retention or weight gain, given their association with approximately 8% to 10% rates of weight gain and edema^[53]^. For resmetirom, gastrointestinal adverse events are the most commonly reported, with similar rates of serious adverse events across treatment arms^[19]^. Cardiac safety in patients with liver disease and ongoing alcohol use remains insufficiently defined. A secondary analysis including 75 patients with “possible MetALD,” defined by elevated carbohydrate-deficient transferrin (CDT) > 2.5% or PEth > 20 ng/mL, demonstrated similar treatment responses at 52 weeks, regardless of alcohol use, suggesting potential efficacy and safety across the MASLD-MetALD spectrum^[90]^.

GLP-1RAs have been repurposed for patients with MetALD. Its safety has been well-established in both MASLD and AUD. Adverse effects are predominantly related to the gastrointestinal tract, including nausea, vomiting, constipation, gastroparesis, and possibly gallbladder-associated adverse effects^[91]^. Further, the GI side effects of GLP-1RA can potentially compound similar manifestations due to ongoing alcohol use. This potential interaction remains unstudied, and warrants caution when prescribing. It should also be recognized that 30%-40% of weight loss is lean body mass and muscle. This is especially relevant in older individuals and patients with cirrhosis, with a risk of obese sarcopenia in those with MASH. In a phase 2 trial, patients receiving semaglutide alone showed a 7.4% reduction in lean mass, compared with 2.9% in patients receiving adjuvant bimagrumab, a monoclonal antibody targeting type II activin receptors and stimulating muscle growth (*P* < 0.001). Similarly, preliminary results from the phase 2 COURAGE trial (NCT06299098) showed that the combination of trevogrumab, an anti-myostatin antibody, with semaglutide reduced lean mass loss by about 50%^[92]^. There were no safety concerns in either study.

Use of GLP-1 agonists requires caution in older adults and patients with cirrhosis, because loss of lean body mass may worsen sarcopenia. Agents targeting *de novo* lipogenesis, such as firsocostat, have shown limited efficacy and have been associated with pruritus and nausea, raising additional tolerability concerns in MetALD^[44,93]^. FXR-based therapy also warrants caution, given the experience with obeticholic acid, which was associated with severe pruritus, an adverse lipid profile, and serious liver-related safety concerns in clinical use, leading to its discontinuation in 2025.

Combining pharmacotherapy for CMRF with FDA-approved MAUD therapy may increase the risk of drug-drug interactions and require closer monitoring. In general, acamprosate is safe in patients with ALD. Naltrexone is metabolized in the liver and carries a black box warning for use in patients with decompensated liver disease, which was removed by the FDA in 2013 (https://wayback.archive-it.org/7993/20161023084600/http://www.fda.gov.laneproxy.stanford.edu/Safety/MedWatch/SafetyInformation/ucm374896.htm)^[94,95]^. Emerging retrospective data suggest that naltrexone may be safe in patients with ALD, including those with cirrhosis. Studies have reported improvements in liver enzymes, lower mortality, and fewer cases of hepatic decompensation among treated patients. In a large VA cohort of patients with cirrhosis and prior decompensation, naltrexone showed an acceptable safety profile^[95]^. For practicing providers, we suggest that naltrexone can be used in patients with ALD without cirrhosis and those with compensated cirrhosis. However, it should be avoided in patients with decompensation, especially those with Child C cirrhosis. Close monitoring is recommended using liver chemistries and clinical evaluation, with the first evaluation at 2-4 weeks after starting naltrexone. The drug should be discontinued if the liver enzymes increase > 3× ULN, bilirubin gets elevated, or a decompensation event develops. Although baclofen, gabapentin, and topiramate lack significant hepatic metabolism, these drugs require dose adjustment based on renal function.

## CONCLUSION

MetALD represents a major unmet need in hepatology, and current management relies largely on extrapolation from separate MASLD/MASH and ALD trials that systematically excluded patients with overlapping metabolic dysfunction and clinically meaningful alcohol exposure. Among emerging therapeutic candidates, GLP-1 receptor agonists and FGF21 analogs appear particularly promising due to their metabolic and antifibrotic benefits, along with a potential for improving alcohol-related behavior. Future MetALD-specific clinical trials should focus on a study population characterized using self-reported alcohol use information complemented by alcohol biomarkers like phosphatidylethanol (PEth); (b) stratification by alcohol exposure, fibrosis stage, and cardiometabolic burden; (c) incorporate integrated liver, alcohol use, metabolic, clinical, and patient-centered outcomes, with focus on validating these end-points in this specific disease; and (d) recruitment and retention strategies.

## Figures and Tables

**Table 1.** Ongoing clinical trials in MASH with potential relevance to MetALD*

Drug class	Investigational agent	NCT number	Phase	Cirrhosis (F4)	Primary endpoint(s)
Incretin (GLP-1)^**^	Survodutide	NCT06632457	Phase 3	Yes	Time to first occurrence of any component of the composite clinical endpoint (all-cause mortality, liver transplant, hepatic decompensation events, worsening MELD to ≥ 15, or progression to CSPH)
Incretin (GLP-1)^**^	Survodutide	NCT06632444	Phase 3	No	Resolution of MASH without worsening of liver fibrosis; ≥ 1-point improvement in fibrosis stage without worsening of MASH (Week 52) Time to first occurrence of any component of the composite clinical endpoint (all-cause mortality, liver transplant, hepatic decompensation events, worsening MELD to ≥ 15, or progression to CSPH)
Incretin^**^ (GIP/GLP-1)	Tirzepatide retatrutide	NCT07165028	Phase 3	No	Time to first occurrence of any component of the composite endpoint for MALO
FGF21^**^	Efruxifermin	NCT06161571	Phase 3	No	Incidence of adverse events and safety outcomes over 52 weeks. Changes in clinical and laboratory parameters over week 52
FGF21^**^	Efruxifermin	NCT06528314	Phase 3	Yes	Time from randomization to first occurrence of disease progression as measured by a composite of protocol-specified clinical events in 5 years Cohort 1 only: ≥ 1-stage improvement in fibrosis with no worsening of steatohepatitis at 96 weeks
FGF21^**^	Pegozafermin	NCT06419374	Phase 3	Yes	Time to first occurrence of disease progression based on composite clinical events; fibrosis regression by ≥ 1 stage at Month 24 Time to occurrence of disease progression upto 5 years
FGF21^**^	Pegozafermin	NCT06318169	Phase 3	No	Co-primary endpoints at Week 52: fibrosis improvement by ≥ 1 stage without worsening of MASH; MASH resolution without worsening of fibrosis
PPAR agonist	Lanifibranor	NCT04849728	Phase 3	No	MASH resolution without worsening of fibrosis; fibrosis improvement by ≥ 1 stage without worsening of MASH week 72
THR-β	Resmetirom (MAESTRO- NASH OUTCOMES)	NCT05500222	Phase 3 (ongoing)	Yes	Incidence of adjudicated composite clinical outcome event (all-cause mortality, liver transplant, ascites, hepatic encephalopathy, gastroesophageal variceal hemorrhage, or MELD increase from < 12 to ≥ 15 due to liver disease)
SGLT2	Empagliflozin	NCT06117137	Phase 3	No	Number of participants with treatment-related adverse events (CTCAE v4.0); number of participants with tightly controlled diabetes mellitus (6 months)
Incretin^**^ (GCG/GLP-1)	Efinopegdutide	NCT04505436	Phase 2	No	Resolution of MASH with no worsening of fibrosis
Incretin^**^ (GCG/GLP-1)	Efinopegdutide	NCT06465186	Phase 2	Yes	Change from baseline in LFC at Week 28; Adverse events up to week 36 and discontinuation rates up to week 28
Incretin^**^ (GCG/GLP-1)	AZD9550 AZD6234	NCT06151964	Phase 2	No	Number and percentage of participants with any adverse event, serious adverse events, or adverse events leading to discontinuation upto day 205
FGFR and VEGFR inhibitors	BI 3802876	NCT07325526	Phase 2	Yes	Occurrence of any AEs up to 134 days
THR-β agonist	HSK31679	NCT06168383	Phase 2	No	Proportion of patients with MASH resolution (2-point reduction in NAS with at least 1-point reduction in ballooning and no increase in steatosis) and no worsening of fibrosis at Week 52
THR-β agonist	ASC 41	NCT05462353	Phase 2	No	Reduction in liver fat content, typically measured via MRI-PDFF, alongside safety and tolerability assessments
Genetic (RNAi)	ALN-HSD-HSD17B13-RNAi	NCT05519475	Phase 2	No	Change in the continuous qFibrosis score measured by second harmonic generation/two-photon excitation microscopy up to week 52
HSD17B13^**^	GSK4532990	NCT05583344	Phase 2	Both F3-F4	Percentage of participants achieving ≥ 1-stage improvement in histologic fibrosis with no worsening of NASH; percentage achieving NASH resolution with no worsening of fibrosis (F3 cohort, Week 52)
ACE inhibitor	Lisinopril	NCT04550481	Phase 2	No	Change in PRO-C3 values week 24
PDE inhibitor	ZSP1601	NCT05692492	Phase 2	No	Resolution of MASH with no worsening of fibrosis; fibrosis regression by ≥ 1 stage
Fibrate	Pemafibrate	NCT06623539	Phase 2	No	Change in ALT week 24
PNPLA3	LY3849891	NCT05395481	Phase 1	No	Number and percentage of participants with any adverse event week 26 Mean change from baseline on liver inflammation and fibrosis content measured by MRI at 24 weeks
Anti-TL1A antibody	Afimkibart, (RO7790121)	NCT06903065	Phase 1	Yes	Percentage of participants with AEs up to Week 52

* These trials are conducted in MASH populations. Given shared pathophysiological mechanisms (metabolic dysfunction, inflammation, and fibrogenesis), these agents may have potential applicability to MetALD; however, dedicated MetALD-specific data are currently limited;

** These trials were conducted in MASH patients and have potential for future metALD trials.

MASH: Metabolic dysfunction-associated steatohepatitis; MetALD: metabolic dysfunction-associated alcohol-related liver disease; GLP-1: glucagon-like peptide-1; GIP: glucose-dependent insulinotropic polypeptide; GCG: glucagon; FGF21: fibroblast growth factor 21; PPAR: peroxisome proliferator-activated receptor; THR-β: thyroid hormone receptor beta; SGLT2: sodium-glucose cotransporter 2; FGFR: fibroblast growth factor receptor; VEGFR: vascular endothelial growth factor receptor; RNAi: RNA interference; HSD17B13: hydroxysteroid 17-beta dehydrogenase 13; PNPLA3: patatin-like phospholipase domain-containing protein 3; TL1A: tumor necrosis factor-like cytokine 1A; NCT: National Clinical Trial identifier; MALO: major adverse liver outcomes; MELD: Model for End-Stage Liver Disease; CSPH: clinically significant portal hypertension; NAS: NAFLD activity score; LFC: liver fat content; AEs: adverse events; CTCAE: Common Terminology Criteria for Adverse Events; qFibrosis: quantitative liver fibrosis.

**Table 2.** Combination pharmacotherapy trials in MASH: key drug classes, regimens, and clinical trial phases

Drug class/Combination	Investigational agents	NCT number	Study phase
SGLT2 + PPAR ± Metformin	Empagliflozin + Pioglitazone + Metformin	NCT04976283	Phase 4/real world
SGLT2 + GLP-1	Empagliflozin + Semaglutide	NCT04639414	Phase 4/real world
Incretins comparators	Tirzepatide *vs*. Retatrutide (Master protocol)	NCT07165028	Phase 3
FGF21 + GLP-1	Zalfermin (NNC0194-0499) + Semaglutide	NCT05016882	Phase 2
PPAR + SGLT2	Lanifibranor + Empagliflozin	NCT05232071	Phase 2
GLP-1 + FXR + ACC	Semaglutide + Cilofexor + Firsocostat	NCT04971785	Phase 2
ASK1 + FXR + ACC	Selonsertib + Cilofexor + Firsocostat	NCT03449446	Phase 2

MASH: Metabolic dysfunction-associated steatohepatitis; ACC: acetyl-CoA carboxylase; ASK1: apoptosis signal-regulating kinase 1; FGF21: fibroblast growth factor 21; FXR: farnesoid X receptor; GLP-1: glucagon-like peptide-1 receptor agonist; NCT: National Clinical Trial identifier; PPAR: peroxisome proliferator-activated receptor; SGLT2: sodium-glucose cotransporter 2.

**Table 3.** Investigational agents in MetALD: active trials and mechanistic targets

NCT number	Phase	Drug	Mechanism tested	Completion	Primary endpoint(s)
NCT06613698	2	GSK4532990	siRNA targeting HSD17B13 to reduce expression of a hepatocyte droplet protein linked to alcohol related and metabolic liver injury	Mar 30, 2028	Number of participants with AEs/SAEs (up to 8 weeks) Clinically relevant ECG/vital sign/laboratory changes (up to 8 weeks) Change from baseline in liver stiffness by FibroScan at week 28; change from baseline in MELD score at week 28
NCT07046819	2	Tirzepatide	Dual GLP-1/GIP receptor agonist aimed at reducing body weight, liver steatosis, and possibly alcohol related behaviors in MetALD	Jul 30, 2026	Metabolic improvement from baseline measured by percentage reduction in body weight Reduction in liver steatosis measured by FibroScan CAP score
NCT07024212	2	DR10624 injection	Long-acting tri-agonist targeting FGF21R, GLP-1R, and GCGR to improve steatosis, insulin sensitivity and weight regulation	Jun 30, 2026	Relative percentage changes (%) of LFC from baseline to Week 12, assessed by MRI-PDFF

MetALD: Metabolic dysfunction-associated alcohol-related liver disease; NCT: National Clinical Trial identifier; AE: adverse event; CAP: controlled attenuation parameter; ECG: electrocardiogram; GCGR: glucagon receptor; GIP: glucose-dependent insulinotropic polypeptide; GLP-1: glucagon-like peptide-1; HSD17B13: hydroxysteroid 17-beta dehydrogenase 13; MRI-PDFF: magnetic resonance imaging proton density fat fraction; MELD: model for end-stage liver disease; SAE: serious adverse event; LFC: liver fat content.

**Table 4.** Alcohol-directed pharmacological therapies for MetALD

	Drugs	Therapeutic effect	Comments
Medications for alcohol use disorder			
	Acomprosate 61-63, 65	Reduces heavy drinking manages cravings during abstinence	NNT =11 to prevent return to any drinking Caution with renal disease
FDA approved medications	Disulfiram	Blocks breakdown of alcohol	Not recommended for use in liver disease
	Naltrexone 62, 64 (oral and Inj)	Reduces heavy drinking Manages cravings	NNT = 18 for oral naltrexone Interaction with opioids
	Topiramate 61, 63	2nd line treatment	Caution in hepatic encephalopathy
	Gabapentin 61-63	2nd line treatment	Caution in hepatic encephalopathy
Non-FDA approved	Varenicline 61-63	Not studies in ALD	Emerging evidence to support its use
medications	Baclofen 61-63	Not FDA approved	Can be used safely in ALD
	Nalmefene 61, 64	Not FDA approved	Reduce alcohol consumption in combination with psychological support
Dual targets			
	GLP-1 RA (Ozempic) 66, 67	Improves metabolic dysfunction and reduces cravings	Phase 2 and phase 3
	Fibroblast growth factor 21 (FGF21) analogues 72, 73	Experimental, reduces alcohol intake in primates	Preclinical
Experimental therapies			
	Fecal Microbiota Transplantation 85, 86	Investigational	Phase 1 and phase 2
	Gene editing therapy (TALENs and CRISPR/Cas9) 83, 86	Under investigation	

ALD: Alcohol-associated liver disease; CRISPR: clustered regularly interspaced short palindromic repeats; FDA: U.S. Food and Drug Administration; FGF21: fibroblast growth factor 21; GLP-1 RA: glucagon-like peptide-1 receptor agonist; Inj: injection; MetALD: metabolic dysfunction-associated alcohol-related liver disease; NNT: number needed to treat; TALENs: transcription activator-like effector nucleases.

**Table 5.** Integrated management framework for MetALD: lifestyle, AUD treatment, and metabolic therapies

Category	Key interventions	Outcomes
Lifestyle and behavioral	Mediterranean/energy-restricted diets; 150-240 min/week moderate-vigorous aerobic exercise; resistance training; bariatric/metabolic surgery when appropriate	≥ 7%-10% weight loss is central; benefits even without weight loss; integrated 24-h lifestyle approach; bariatric surgery reduces intrahepatic fat and NAS and improves fibrosis
Manage cardio-metabolic risk	Aggressive management of diabetes, dyslipidemia, hypertension, obesity and OSA; statins; GLP-1 RAs and SGLT-2 inhibitors	Optimizing blood pressure, lipids and glycaemia reduces cardiovascular risk; statins safe across MASLD spectrum; screen and treat OSA; GLP-1 RAs/SGLT-2 inhibitors improve steatosis, weight and cardiovascular outcomes
AUD pharmacotherapy	Structured psychosocial interventions; acamprosate; naltrexone; consider baclofen, gabapentin, topiramate	Acamprosate increases abstinence and lacks hepatic metabolism; naltrexone reduces heavy drinking but avoid in Child-Pugh C; hepatic safety study showed decreased ALT/AST after naltrexone; baclofen and others provide alternatives
Metabolic pharmacotherapy	Resmetirom (THR-β agonist); GLP-1 RAs (semaglutide, liraglutide); SGLT-2 inhibitors	Resmetirom achieved NASH resolution in 25.9%-29.9% and improved fibrosis; GLP-1 RA trial showed 59% NASH resolution but no significant fibrosis improvement. GLP-1RA also improve weight, glycemic control and lipids
Microbiome and adjunctive	Faecal microbiota transplantation; probiotics; prebiotics	Consecutive FMT did not improve liver fat or metabolic parameters at 12 weeks; microbiome therapies remain experimental

ALT: Alanine aminotransferase; AST: aspartate aminotransferase; AUD: alcohol use disorder; FMT: fecal microbiota transplantation; GLP-1 RA: glucagon-like peptide-1 receptor agonist; MASLD: metabolic dysfunction-associated steatotic liver disease; MetALD: metabolic dysfunction-associated alcohol-related liver disease; NASH: nonalcoholic steatohepatitis; NAS: NAFLD activity score; OSA: obstructive sleep apnea; SGLT-2: sodium-glucose cotransporter 2; THR-β: thyroid hormone receptor beta.

## Data Availability

Not applicable.
